# A High Protein Diet Has No Harmful Effects: A One-Year Crossover Study in Resistance-Trained Males

**DOI:** 10.1155/2016/9104792

**Published:** 2016-10-11

**Authors:** Jose Antonio, Anya Ellerbroek, Tobin Silver, Leonel Vargas, Armando Tamayo, Richard Buehn, Corey A. Peacock

**Affiliations:** Exercise and Sport Science Laboratory, Nova Southeastern University, Davie, FL, USA

## Abstract

The purpose of this investigation was to determine the effects of a high protein diet over a one-year period. Fourteen healthy resistance-trained men completed the study (mean ± SD; age 26.3 ± 3.9 yr; height 178.5 ± 8.4 cm; and average years of training 8.9 ± 3.4 yr). In a randomized crossover design, subjects consumed their habitual or normal diet for 2 months and 4 months and alternated that with a higher protein diet (>3 g/kg/d) for 2 months and 4 months. Thus, on average, each subject was on their normal diet for 6 months and a higher protein diet for 6 months. Body composition was assessed via the Bod Pod**®**. Each subject provided approximately 100–168 daily dietary self-reports. During the subjects' normal eating phase, they consumed (mean ± SD) 29.94 ± 5.65 kcals/kg/day and 2.51 ± 0.69 g/kg/day of protein. This significantly increased (*p* < 0.05) during the high protein phase to 34.37 ± 5.88 kcals/kg/day and 3.32 ± 0.87 g/kg/day of protein. Our investigation discovered that, in resistance-trained men that consumed a high protein diet (~2.51–3.32 g/kg/d) for one year, there were no harmful effects on measures of blood lipids as well as liver and kidney function. In addition, despite the total increase in energy intake during the high protein phase, subjects did not experience an increase in fat mass.

## 1. Introduction

It has been postulated that the consumption of a high protein diet may cause harmful effects, particularly in the kidneys. Approximately a century ago, investigators found “at least some or very severe” renal damage in a small group of rats on a high protein diet in which one kidney had been removed [1]. Other work on rodents found no evidence of renal damage; however, they did find that rats receiving a high protein diet experienced renal hypertrophy [2]. Notwithstanding, a more recent rat study reported that 30 days of very high whey protein supplemented diet (i.e., 6 human-equivalent 20 g doses per day) did not adversely affect blood and/or histological markers of liver or kidney health and instead may improve liver health when compared to rats not receiving protein [3].

The challenge with determining the effects of high protein diets on measures of health is the lack of agreement with what constitutes a “high” intake of protein. At least in athletic populations, the International Society of Sports Nutrition's® position stand on protein states that “protein intakes of 1.4–2.0 g/kg/day for physically active individuals is not only safe, but may improve the training adaptations to exercise training” [4]. Furthermore, scientists have used different definitions of “high” protein intakes. For instance, protein intakes greater than 15-16% of total energy, as high as 35% of total calories, or intakes that exceed the RDA have been postulated as reaching the threshold of what constitutes a “high protein” diet [5]. We would posit that basing a diet on percentages is misleading. That is, if one were to consume a hypoenergetic diet of 1000 kcal in which 35% of the calories were derived from protein, then that would amount to a paltry 87.5 grams of protein. Instead, high protein diets should always be defined as the amount of protein consumed per unit body weight. It is our contention that high protein diets should necessarily exceed 2.0 g/kg/d. Previous work from our laboratory discovered that an eight-week period of heavy resistance training coupled with high protein consumption (>3.0 g/kg/d) results in improvements in body composition [6]. Furthermore, at least in the short term, high protein intakes had no harmful side effects [6, 7]. However, long-term longitudinal data are lacking in terms of the effects of high protein diets. Thus, the purpose of this investigation was to examine the effects of high protein consumption in a group of resistance-trained young males over a 1-year period.

## 2. Methods and Materials

### 2.1. Participants

Fourteen resistance-trained male subjects volunteered for this investigation (racial/ethnic background: 10 white males, 3 black males, and 1 Pacific Islander). Subjects took part in a randomized crossover trial in which they consumed their habitual (i.e., normal protein) or high protein diet for two months and four months, respectively. The order in which they consumed their normal or high protein intakes was randomized. Subjects followed their normal and high protein intake phases for a total of 6 months, respectively. Subjects came to the laboratory on five occasions. They were tested at baseline and then subsequently after two 2-month periods and two 4-month periods of following the respective diet. The extra protein consumed by each subject was obtained primarily from whey protein powder, which was provided to each subject at no cost (Dymatize**®** ISO-100 with 25 grams of protein, 1 gram of carbohydrate, and zero grams of fat per serving of one scoop). However, subjects did not have to consume the extra protein as powder; instead, they could consume whatever extra protein source they preferred. Nova Southeastern University's Human Subjects Institutional Review Board in accordance with the Helsinki Declaration approved this study and written informed consent was obtained prior to participation.

### 2.2. Food Diary

Subjects kept a diary (i.e., three days per week for one year) of their food intake via a smartphone app (MyFitnessPal) equaling ~150 daily food logs over the treatment period. The use of mobile apps for dietary self-reporting has been previously used [6–9]. Every subject had previous experience with this mobile app. The MyFitnessPal app is a database comprised of over 5 million foods that have been provided by users via entering data manually or by scanning the bar code on packaged goods. Thus, the data themselves are primarily derived from food labels (i.e., nutrition facts panel) derived from the USDA National Nutrient database.

### 2.3. Body Composition

Height was measured using standard anthropometry and total body weight was measured using a calibrated scale. Body composition was assessed by whole body densitometry using air displacement via the Bod Pod (COSMED USA, Concord, CA). All testing was performed in accordance with the manufacturer's instructions. Subjects were instructed to come into the lab after a 3-hour fast and no exercise 24 hours prior to assessment. They voided prior to testing. Subjects were tested while wearing only tight fitting clothing (swimsuit or undergarments) and an acrylic swim cap. Subjects were instructed to wear the same clothing for all testing. Thoracic gas volume was estimated for all subjects using a predictive equation integral to the Bod Pod software. Each subject was tested at least twice per visit. Data from the Bod Pod include body weight, percent body fat, fat-free mass, and fat mass. All testing was done with each subject at approximately the same time of day for each of the five testing sessions. Although hydration status was not assessed, each subject was tested in an identical manner throughout the investigation. The Bod Pod was calibrated the morning of the testing session as well as between each subject.

### 2.4. Blood Analysis: Comprehensive Metabolic Panel and Blood Lipids

Subjects presented in a fasted state at a local Quest Diagnostics™ facility on five separate occasions. A blood lipid and comprehensive metabolic panel was done. This includes the following measures: glucose, blood urea nitrogen (BUN), creatinine, glomerular filtration rate, BUN/creatinine ratio, sodium, potassium, chloride, carbon dioxide, calcium, total protein, albumin, globulin, albumin/globulin ratio, total bilirubin, alkaline phosphatase, alanine transaminase, aspartate transaminase, total cholesterol, high-density lipoprotein cholesterol, triglycerides, low-density lipoprotein cholesterol, and the total cholesterol to high-density lipoprotein cholesterol ratio. Quest Diagnostics performed each test according to the standard operating procedure of the company.

### 2.5. Training Program

Each subject followed their own strength and conditioning program. The investigators were in regular contact with each subject to ensure that each subject completed a training log. The volume load (i.e., total weight lifted per week) was determined for each treatment period.

### 2.6. Statistics

A 2-way analysis of variance (ANOVA) was used to analyze the data with *p* < 0.05 considered as significant. The data that were compared were baseline and the mean of the normal treatment period [combined 2-month and 4-month treatment] as well as the mean of the high protein treatment period [combined 2-month and 4-month treatment]. Data are expressed as the mean ± SD. The statistical analysis was completed using Prism 6 GraphPad Software (La Jolla, California).

## 3. Results

The data for body composition are shown in Table 1. The data for nutritional intake are shown in Table 2. Subjects consumed more absolute and relative calories and protein during the high protein phase (*p* < 0.05). There were no significant differences between the normal and high groups in any measure of health or body composition (Tables 3 and 4). It should be noted that one subject completed 6 months on the high protein phase and only 2 months on the normal protein phase. He did not complete the final 4 months of the normal protein phase due to geographic relocation. Thus, for this particular subject, we compared the mean of his normal (2 months of data) and high protein phases (6 months of data).

## 4. Discussion

This is the first randomized controlled trial that has examined the effects of a high protein diet in resistance-trained subjects over a 1-year treatment period. In brief, we found no deleterious effects of a high protein diet (2.51–3.32 g/kg/d) over a 1-year period. Prior work from our lab has shown that consuming a high protein diet in the short term has no harmful effects on any clinical measure (i.e., blood lipids and comprehensive metabolic panel) [6, 7].

The subjects in the current investigation alternated between their normal or habitual protein intake and a high protein intake. It should be noted however that even their normal protein intake would be considered high by other investigators [5, 11, 12]. Thus, our study does not support the notion that protein intakes 3-4 times greater than the current RDA cause any harmful effects.

Moreover, the amount of dietary fiber consumed by our subjects was ~30 grams per day. This is in contrast with the average fiber intake in the United States of ~16 grams per day [13]. Thus, it is a falsehood to promote the idea that high protein diets are mutually exclusive with a diet that is also high in fiber. Our subjects showed no harmful effects of a hyperenergetic, high protein diet and this (i.e., blood lipids, renal and hepatic function, etc.) may have been due partially to their fiber intake. It is known that higher fiber intakes are associated with a lower risk of cardiovascular disease, cancer, and all-cause mortality [14–16]. On the other hand, the cholesterol intake of our subjects was twice as high as the typical recommendation of 300 mg per day [17]. The notion that high cholesterol intakes have a deleterious effect on blood lipid markers of cardiovascular disease is not supported by our data.

Prior work from our laboratory has shown that consuming protein (2.3–3.4 g/kg/d) in amounts that are 3-4 times greater than the RDA results in a similar FFM increase for both the normal and high protein groups [6]; however, the high protein group lost more fat mass compared to the normal protein group in spite of the fact that they consumed on average ~400 kcals more per day over the treatment period. This is in contrast with the current study that showed no change in body composition. The primary difference between the current study and the aforementioned one is that subjects in the current study did not purposely alter their training program. On the other hand, subjects in our previous study were subjected to a different training stimulus (i.e., periodized resistance-training program) than they had been accustomed to. Inasmuch as the focus on our current work was on the markers of health, subjects in the current study were instructed to not alter their training regimen. An examination of their volume load shows indeed that they did not make any significant alterations in training volume. Thus, one would speculate that, without significant changes in the training stimulus, the mere provision of extra protein would likely not lead to changes in body composition. Conversely, the mere addition of extra protein calories also will not lead to gains in fat mass.

### 4.1. Limitations

One might speculate that a limitation of this investigation is that the subjects were young males who had several years of resistance-training experience and were regularly consuming a high protein diet at baseline. However, the fact that they increased their protein intake by ~32% and still had no deleterious side effects is further evidence that a high protein diet in exercise-trained individuals is indeed safe. The small sample size may also preclude one from applying the results from this study to other populations (i.e., sedentary men or women). Nonetheless, we would posit that the only populations that would consume a high protein diet are athletes (e.g., highly trained endurance and strength-power athletes). Thus, the need to apply our data to other populations may be a moot point.

## 5. Conclusions

In male subjects with several years of experience with resistance training, chronic consumption of a diet high in protein had no harmful effects on any measures of health. Furthermore, there was no change in body weight, fat mass, or lean body mass despite eating more total calories and protein. Contrary to popular belief, the consumption of a high protein diet is not mutually exclusive with a diet high in fiber nor does the consumption of cholesterol above the standard recommendations result in any untoward effects on blood lipids. This is the first 1-year longitudinal investigation in resistance-trained males that demonstrates the lack of harm caused by a high protein diet.

## Figures and Tables

**Table 1:** Body composition and training volume.

	Baseline	Normal	High
Weight kg	84.05 ± 10.20	84.55 ± 10.73	85.18 ± 11.04
Fat mass kg	11.42 ± 3.13	12.39 ± 3.85	12.30 ± 3.26
FFM kg	72.63 ± 9.19	72.15 ± 8.41	72.73 ± 9.65
% body fat	13.59 ± 3.21	14.48 ± 3.48	14.49 ± 3.59
Volume load^*∗*^	50,160 ± 17,510	48,684 ± 19,436	51,023 ± 20224

Data are mean ± SD. There were no significant differences between groups. ^*∗*^Volume load is equal to the mean total amount of weight lifted (kg) each week.

**Table 2:** Dietary intake.

	Baseline	Normal	High
Kcal	2452 ± 571	2511 ± 479	2919 ± 562^*∗*^
CHO g	229 ± 75	226 ± 64	241 ± 71
PRO g	196 ± 92	214 ± 76	284 ± 90^*∗*^
Fat g	83 ± 35	83 ± 22	91 ± 23

Kcal/kg/d	29.40 ± 7.19	29.94 ± 5.65	34.37 ± 5.88^*∗*^
CHO g/kg/d	2.78 ± 0.94	2.74 ± 0.89	2.87 ± 0.94
PRO g/kg/d	2.31 ± 1.03	2.51 ± 0.69	3.32 ± 0.87^*∗*^
Fat g/kg/d	1.00 ± 0.41	0.99 ± 0.26	1.07 ± 0.23
Cholesterol mg	535 ± 336	425 ± 242	602 ± 310
Sodium mg	3042 ± 1360	3228 ± 1069	3562 ± 964
Sugars g	55 ± 38	56 ± 22	62 ± 22
Fiber g	26 ± 15	29 ± 13	31 ± 13

Data are mean ± SD. ^*∗*^Significant difference (baseline versus high and normal versus high; *p* < 0.05).

CHO: carbohydrate, d: day, g: gram, kcal: calorie, kg: kilogram, and PRO: protein.

**Table 3:** Blood lipids.

	Baseline	Normal	High	Reference range
Total cholesterol mg/dL	158 ± 28	146 ± 21	151 ± 26	125–200
HDL-C mg/dL	48 ± 15	48 ± 12	45 ± 11	> or = 40
TG mg/dL	61 ± 18	62 ± 24	64 ± 18	<150
LDL-C mg/dL	98 ± 33	86 ± 18	92 ± 22	<130
Cholesterol/HDL-C ratio	3.9 ± 3.0	3.5 ± 1.6	3.5 ± 0.9	< or = 5.0

Data are mean ± SD. There were no significant differences between groups. C: cholesterol, dL: deciliter, HDL: high-density lipoprotein, LDL: low-density lipoprotein, mg: milligram, and TG: triglycerides.

**Table 4:** Comprehensive metabolic panel.

	Baseline	Normal	High	Reference range
Glucose mg/dL	84 ± 11	87 ± 13	85 ± 9	65–99
BUN mg/dL	22 ± 6	22 ± 5	22 ± 4	7–25
Creatinine mg/dL	1.1 ± 0.2	1.1 ± 0.1	1.1 ± 0.2	0.60–1.35
eGFR	95 ± 19	101 ± 17	98 ± 16	#
BUN/creatinine ratio	20 ± 5	21 ± 5	20 ± 3	6–22
Sodium mmol/L	139 ± 2	139 ± 1	138 ± 1	135–146
Potassium mmol/L	4.3 ± 0.4	4.4 ± 0.3	4.3 ± 0.2	3.5–5.3
Chloride mmol/L	103 ± 1.7	102 ± 1.5	102 ± 1.7	98–110
CO_2_ mmol/L	28 ± 2	28 ± 2	28 ± 2	19–30
Calcium mg/dL	9.6 ± 0.2	9.6 ± 0.2	9.7 ± 0.3	8.6–10.3
Total protein g/dL	7.2 ± 0.3	7.1 ± 0.4	7.1 ± 0.3	6.1–8.1
Albumin g/dL	4.6 ± 0.2	4.5 ± 0.2	4.5 ± 0.2	3.6–5.1
Globulin g/dL	2.5 ± 0.3	2.6 ± 0.3	2.6 ± 0.3	1.9–3.7
Alb/Glob ratio	1.8 ± 0.2	1.8 ± 0.2	1.8 ± 0.2	1.0–2.5
Total Bili mg/dL	0.7 ± 0.4	0.8 ± 0.6	0.7 ± 0.3	0.2–1.2
Alkaline phosphatase U/L	64 ± 17	67 ± 17	66 ± 16	40–115
AST U/L	28 ± 9	27 ± 6	31 ± 13	10–40
ALT U/L	28 ± 18	26 ± 8	31 ± 15	9–46

Data are mean ± SD. There were no significant differences between groups. Alb: albumin, ALT: alanine transaminase, AST: aspartate transaminase, Bili: bilirubin, BUN: blood urea nitrogen, eGFR: estimated glomerular filtration rate, g: grams, Glob: globulin, mmol: millimoles, L: liter, and mg: milligrams. # indicates a value > or = 60 mL/min/1.73 m^2^.
